# Entropy in Foundations of Quantum Physics

**DOI:** 10.3390/e22030371

**Published:** 2020-03-24

**Authors:** Marcin Pawłowski

**Affiliations:** International Centre for Theory of Quantum Technologies, University of Gdańsk, 80-952 Gdańsk, Poland; marcin.pawlowski@ug.edu.pl

**Keywords:** foundations of quantum mechanics, quantum cryptography, entropy

Entropy can be used in studies on foundations of quantum physics in many different ways, each of them using different properties of this mathematical object. First of all, entropy can be intuitively understood and we can exploit that fact by finding ways to derive predictions of quantum mechanics without employing the full mathematical apparatus of that theory. Instead, we can propose operational axioms which we can more easily understand and try to find the reasons why the universe behaves in the way that it does.

The second reason for its usefulness stems simply from how convenient it is to use entropy in different aspects of information processing. It is therefore an indispensable tool for quantum information theory, which recently has been the field that led to the most breakthroughs in foundations of physics. 

Finally, sheer ubiquity of entropy in physics and other fields makes it a possible bridge between different areas, enabling us to carry insights from one to another. 

In this Special Issue, we find examples of papers which employ each of these approaches. 

In the paper “Hypergraph Contextuality” [1], the author introduces a new form of quantum contextuality. The two previously known forms were Kochen–Specker (KS) [2] and observable-based [3] contextualities. In paper [1], hypergraphs with 3-dim vectors are considered, in which some of those vectors that belong to only one triplet are dropped, as in the observable approach, and smaller hypergraphs are generated from them, such that one cannot assign definite binary values to them, as in the KS approach. This new approach is called hypergraph contextuality and allows us, among other things, to establish new entropic contextualities.

In the paper “The Entropic Dynamics Approach to Quantum Mechanics” [4], the author develops his theory of Entropic Dynamics introduced in [5,6,7]. In this paper [4], A new version of Entropic Dynamics is introduced in which particles follow smooth differentiable Brownian trajectories in order to discuss why wave functions are complex and the connections between the superposition principle, the single-valuedness of wave functions, and the quantization of electric charges.

In the paper “A New Mechanism of Open System Evolution and Its Entropy Using Unitary Transformations in Noncomposite Qudit Systems” [8], the authors develop further their method introduced in [9], which models the dynamics of open system evolution of qubits by the unitary evolution of qutrits instead of by composite systems as it is usually done. In particular, they apply their methodology to study the behavior of phase damping and spontaneous emission channels and compute the evolution of the state’s entropy in these channels.

In the paper “Uniqueness of Minimax Strategy in View of Minimum Error Discrimination of Two Quantum States” [10], the authors consider minimum error discrimination of two quantum sates as a game. This is not a new approach; however, in this paper [10], it is generalized to take into account different prior probabilities for the states, choosing which constitutes the sender’s strategy. They are able to obtain the necessary and sufficient condition for the uniqueness of it. They also provide a condition for when the sender’s minimax strategy and the receiver’s optimal minimum error strategy cannot both be unique.

Paper [11] deals with the issue of parameter estimation in continuous variable QKD. This is very simple problem with a straightforward solution if we work in an asymptotic limit. This is, however, not very practical and if one considers realistic, finite-size scenario, the case becomes more complex. Still, the authors of [11] have been able to adapt the parameter estimation technique to the entropic uncertainty relation analysis method under composable security frameworks. Moreover, in their approach, all the states can be exploited for both parameter estimation and key generation.

In the paper “On the Exact Variance of Tsallis Entanglement Entropy in a Random Pure State” [12], the author studies the variance of the Tsallis entropy of bipartite quantum systems in a random pure state. He is able to obtain an exact variance formula of the Tsallis entropy that involves finite sums of some terminating hypergeometric functions, which in some cases can be simplified to more compact equations.

In the paper “Probabilistic Resumable Quantum Teleportation of a Two-Qubit Entangled State” [13], the authors introduce resumable quantum teleportation of a two-qubit, entangled, pure state. Resumable here refers to the fact that the entanglement shred between the parties does not allow for perfect deterministic teleportation, so the protocol sometimes fails. However, in these cases, the sender is notified and can recover her initial state and try to teleport again until successful. 

The paper by Jiménez et al. [14] is another paper in this issue that looks at minimum error discrimination. While, in the paper by Kim et al. [10], the authors were studying optimal strategies, Jiménez et al. [14] focuses on discrimination as a process and studies it as a thermodynamic cycle. The authors consider the amount of quantum discord consumed and show that thermal discord is lower than the entropy generated.

One paper which, in my opinion, stands out in this issue is [15]. It is much more philosophical than others and perhaps fits the title “Entropy in Foundations of Quantum Physics” the best. The author deals with different interpretations of quantum mechanics and the whole paper is an extensive defense of a point of view that quantum states codify observer-relative information. The entropy enters here because it is argued that probabilities relative to a non-participating observer evolve according to an entropy maximizing principle.

In the paper “Some Consequences of the Thermodynamic Cost of System Identification” [16], the author studies the problem of system identification. He uses the standard tool of quantum thermodynamics to approach this surprisingly overlooked problem. The main result is the impossibility of arbitrarily precise identification and the links between this process and the violation of CHSH and Leggett-Garg inequalities.

Arguably, one of the most interesting papers in the issue is [17]. Usually, the insights from classical information processing are used to develop foundations of quantum mechanics. Here the ideas from the latter are used in the former. The authors of [17] propose a novel image encoding method inspired by quantum theory, representing the details by density matrices. Then, they can use the techniques for maximization of von Neumann entropy to improve image thresholding.

In the paper “Quantum Quantifiers for an Atom System Interacting with a Quantum Field Based on Pseudoharmonic Oscillator States” [18], the authors develop the Jaynes–Cummings model, considering the interaction between a two-level atom and a quantum field in the framework of pseudoharmonic oscillator potentials. They also qualitatively examined various quantum quantifiers in terms of the initial parameters during time evolution with and without time-dependent coupling, considering the quantum entanglement, geometric phase, nonclassicality and atomic squeezing.

Paper [19] develops the ideas of self-referenced continuous-variable quantum key distribution introduced in [20], which is a Gaussian modulated coherent state-continuous variable protocol with a local oscillator generated at the receiver’s lab. The idea of [19] is to use the virtual photon subtraction method introduced in [21] for this type of Quantum Key Distribution. The authors show that it can lead to greater robustness and longer maximal distances in practical quantum cryptography.

The contribution by Wang et al. [22] is the third paper, after [11] and [19], on continuous variable quantum key distribution. In this paper [22], the authors study a unidimensional version of that protocol. Their main result is that adding optimal noise to the receiver improves the resistance of the protocol to excess noise.

The last, but definitely not least, paper [23] in this issue attempts an explanation of Tsirelson bound via a communication protocol. The authors propose the Statistical No-Signaling principle, which dictates that no information can pass through a disconnected channel. It is very similar in spirit to Information Causality [24], as both deal with information passing through a channel made using van Dam construction [25] and lead to the same restrictions on the maximal quantum violation of CHSH and Uffink inequalities. The main difference between the two principles is that Information Causality provides insights from the theory of communication, while Statistical No-Signaling from statistical inference.

I hope that the papers of this issue will keep the interest in quantum foundations high and inspire even more work in that field in future.

## References

[1] Pavičić M. (2019). Hypergraph Contextuality. Entropy.

[2] Bengtsson I., Blanchfield K., Cabello A. (2012). A Kochen–Specker Inequality from a SIC. Phys. Lett. A.

[3] Yu S., Oh C.H. (2012). State-Independent Proof of Kochen-Specker Theorem with 13 Rays. Phys. Rev. Lett..

[4] Caticha A. (2019). The Entropic Dynamics Approach to Quantum Mechanics. Entropy.

[5] Caticha A. (2011). Entropic Dynamics, Time, and Quantum Theory. J. Phys. A Math. Theor..

[6] Caticha A. (2015). Entropic Dynamics. Entropy.

[7] Caticha A. (2018). Entropic Dynamics: Quantum Mechanics from Entropy and Information Geometry. Ann. Physik.

[8] López-Saldívar J.A., Castaños O., Man’ko M.A., Man’ko V.I. (2019). A New Mechanism of Open System Evolution and Its Entropy Using Unitary Transformations in Noncomposite Qudit Systems. Entropy.

[9] Chernega V.N., Man’ko O.V., Man’ko V.I. (2017). Triangle Geometry of the Qubit State in the Probability Representation Expressed in Terms of the Triada of Malevich’s Squares. J. Russ. Laser Res..

[10] Kim J., Ha D., Kwon Y. (2019). Uniqueness of Minimax Strategy in View of Minimum Error Discrimination of Two Quantum States. Entropy.

[11] Chen Z., Zhang Y., Wang X., Yu S., Guo H. (2019). Improving Parameter Estimation of Entropic Uncertainty Relation in Continuous-Variable Quantum Key Distribution. Entropy.

[12] Wei L. (2019). On the Exact Variance of Tsallis Entanglement Entropy in a Random Pure State. Entropy.

[13] Wang Z.-Y., Gou Y.-T., Hou J.-X., Cao L.-K., Wang X.-H. (2019). Probabilistic Resumable Quantum Teleportation of a Two-Qubit Entangled State. Entropy.

[14] Jiménez O., Solís-Prosser M.A., Neves L., Delgado A. (2019). Quantum Discord, Thermal Discord, and Entropy Generation in the Minimum Error Discrimination Strategy. Entropy.

[15] Krismer R. (2018). Representation Lost: The Case for a Relational Interpretation of Quantum Mechanics. Entropy.

[16] Fields C. (2018). Some Consequences of the Thermodynamic Cost of System Identification. Entropy.

[17] Wang X., Yang C., Xie G.-S., Liu Z. (2018). Image Thresholding Segmentation on Quantum State Space. Entropy.

[18] Raffah B.M., Berrada K. (2018). Quantum Quantifiers for an Atom System Interacting with a Quantum Field Based on Pseudoharmonic Oscillator States. Entropy.

[19] Zhong H., Wang Y., Wang X., Liao Q., Wu X., Guo Y. (2018). Enhancing of Self-Referenced Continuous-Variable Quantum Key Distribution with Virtual Photon Subtraction. Entropy.

[20] Soh D.B.S., Brif C., Coles P.J., Lütkenhaus N., Camacho R.M., Urayama J., Sarovar M. (2015). Self-Referenced Continuous-Variable Quantum Key Distribution Protocol. Phys. Rev. X.

[21] Li Z.-Y., Zhang Y.-C., Wang X.-Y., Xu B.-J., Peng X., Guo H. (2016). Non-Gaussian postselection and virtual photon subtraction in continuous-variable quantum key distribution. Phys. Rev. A.

[22] Wang P., Wang X., Li Y. (2018). Security Analysis of Unidimensional Continuous-Variable Quantum Key Distribution Using Uncertainty Relations. Entropy.

[23] Carmi A., Moskovich D. (2018). Tsirelson’s Bound Prohibits Communication through a Disconnected Channel. Entropy.

[24] Pawlowski M., Paterek T., Kaszlikowski D., Scarani V., Winter A., Zukowski M. (2009). Information causality as a physical principle. Nature.

[25] Van Dam W. (2013). Implausible consequences of superstrong nonlocality. Nat. Comput..

